# The Tell-Tale Look: Viewing Time, Preferences, and Prices

**DOI:** 10.1371/journal.pone.0117137

**Published:** 2015-01-12

**Authors:** Brian C. Gunia, J. Keith Murnighan

**Affiliations:** 1 Johns Hopkins University, Carey Business School, 100 International Drive, 13^th^ Floor, Baltimore, MD 21202, United States of America; 2 Northwestern University, Kellogg School of Management, 2001 Sheridan Road, Evanston, IL 60208, United States of America; University of Verona, ITALY

## Abstract

Even the simplest choices can prompt decision-makers to balance their preferences against other, more pragmatic considerations like price. Thus, discerning people’s preferences from their decisions creates theoretical, empirical, and practical challenges. The current paper addresses these challenges by highlighting some specific circumstances in which the amount of time that people spend examining potential purchase items (i.e., viewing time) can in fact reveal their preferences. Our model builds from the gazing literature, in a purchasing context, to propose that the informational value of viewing time depends on prices. Consistent with the model’s predictions, four studies show that when prices are absent or moderate, viewing time provides a signal that is consistent with a person’s preferences and purchase intentions. When prices are extreme or consistent with a person’s preferences, however, viewing time is a less reliable predictor of either. Thus, our model highlights a price-contingent “viewing bias,” shedding theoretical, empirical, and practical light on the psychology of preferences and visual attention, and identifying a readily observable signal of preference.

## Introduction

Honda, Chevy, Lexus, Ferrari? Americans purchase more than 40 million used cars each year [1], and spend about 73% of their shopping time online [2]. Although the internet obviously makes it easier to review a car’s features and prices, buyers must still integrate this information into a final decision. Indeed, the sheer volume of information on the internet may create additional challenges for car buyers, who, like many other consumers and decision-makers, must often balance their preferences against more pragmatic issues like price. As a result, decision-makers’ final choices often reflect more than their preferences; Honda buyers, for example, may wish they could have bought a Ferrari. Thus, although “revealed preferences” [3] indicate the chosen option, they do not necessarily indicate the most-preferred option, independent of other considerations. In other words, discerning people’s preferences from their decisions—the topic of the current research—poses a particular challenge.

In searching for an alternative indicator of preferences, we move away from a person’s final, revealed preference to focus on elements of their decision-making process. In particular, we focus on an inherent and readily observable signal, viewing time: how long people spend examining potential purchase items. In the age of online purchasing, viewing is a critical step in the decision-making process, and one that “virtually every commercial website monitors” [4] via clickstream data. Since research and theory on the interpretation of viewing time remain relatively thin, though, we use insights from research on other forms of visual attention and choice [5–7], especially recent models of gazing (the direction and duration of eye fixations) [8–10].

A central finding in the gazing literature is the gaze bias: people visually fixate on items that they ultimately choose more than they fixate on items that that they ultimately reject [8, 10]. We know of little research, however, that has connected this pivotal finding to purchasing decisions or to more general and readily observable forms of viewing behavior like viewing time [11]. Additionally, we know of little research that has linked viewing time to preferences in economic decisions. Thus, we extend a recent model of gaze bias [9] into the domain of viewing to build a theoretical foundation for studying the relationship between viewing time and preferences. Our model identifies the particular conditions when viewing time is likely to reveal preferences. In particular, we suggest that the informational value of viewing time depends on prices: viewing time provides diagnostic information on preferences when prices are absent or “moderate” (defined and explained below) but not when they are “extreme” or consistent with an individual’s preferences.

To test these hypotheses, we conducted four experiments that simulated several essential elements of online purchasing decisions. By asking participants to browse through a preselected set of items at their leisure, we controlled the many reasons for real consumer browsing, and also unobtrusively monitored participants’ viewing times [12]. The data consistently support our predictions, highlighting a price-contingent “viewing bias.” Theoretically, the findings help to: 1) reconcile ongoing debates on the source of the gaze bias, 2) integrate three literatures that have developed in relative isolation (gazing, viewing, and purchasing), and 3) provide new insights into the psychology of viewing and decision-making. Empirically, they offer an easy and unobtrusive method for measuring preferences. Practically, they highlight both the value and the limits of the visual attention data that almost every commercial website collects.

## Viewing Time

Many decisions prompt people to balance what they would like to do against what they should do [13]. In the purchasing domain, this often means integrating preferences and price considerations, with prices limiting preferences’ free rein. As a result, purchases and other decisions typically reflect the outcome of this integration process rather than pure preferences *per se*, pushing research that focuses on detecting individuals’ preferences away from decisions and toward decision-making processes [8–10]. In the current research, we focus on viewing time as a critical and readily observable signal of the decision-making process.

In the purchasing domain, people often seek new information on potential purchase items, i.e., they browse the available items [14]. Traditionally, browsing consisted of visiting a store and viewing its products. Online browsing replaces stores with commercial websites, but still consists of visiting and viewing [11]. For online purchases, especially those involving expensive or intangible elements (e.g., an extended cruise), browsing becomes particularly important because online purchasers obtain much of their required information visually [12, 14]. Online or offline, a central indicator of browsing is the time that decision-makers spend viewing potential purchase items.

Of course, viewing time does not always predict purchasing decisions, especially online. Since consumers incur fewer costs from visiting a website than from visiting a store, they tend to browse early and often, for reasons ranging from novelty-seeking to information-gathering to purchasing [12, 14]. Although viewing may not lead to a purchase [15], our research is prefaced on the notion that viewing time correlates positively with both interest [16] and purchases [11]. In particular, consumers who ultimately make a purchase must often start the process by viewing a product or service to learn about it; they later return to weigh economic considerations and decide whether to purchase [17]. We designed four experiments to model this multi-stage viewing / decision process.

Because people view commercial websites for many reasons, cross-sectional research on viewing time has presented difficult analytical and statistical challenges [12]. Thus, although most commercial websites already record clickstream data on viewing time [17], evidence on the psychology of viewing and especially its potential relationship with preferences and prices is slim. Indeed, although several studies have examined viewing behavior in the presence of prices [18–22], we know of none that has attempted to discern pure preferences from viewing time. Thus, we present and test a model that connects viewing time to people’s preferences in purchasing decisions.

Our approach controls the many reasons for browsing by asking all participants to view the same items for the same reasons. Given the obvious importance of discerning what the decision-maker likes *most*, to both the decision-maker and those interested in observing the decision-maker, we focus on what viewing time can say about an individual’s most-preferred option. In other words, we investigate whether, and under what conditions, viewing time can reveal a decision-maker’s most-preferred option vis-à-vis less preferred but potentially more pragmatic options. To develop hypotheses on the signaling value of viewing time, we use insights from the literature that has come closest to considering these issues in the past—the gazing literature.

## Viewing versus Gazing

Research on gazing has capitalized on the common view that people’s eyes act as “windows to the soul,” as well as the scientific insight that “The eyes don’t lie. If you want to know what people are paying attention to, follow what they are looking at” [23]. Thus, it has treated gazing, i.e., the direction and duration of people’s eye fixations on specific physical locations [10], as a critical indicator of attention and choice [24–26]. Gazing is a subset of viewing: people can view a particular product’s webpage without gazing at the product, but they cannot gaze at a product without first viewing its webpage.

This highlights an important theoretical difference between viewing and gazing: whereas gazing is typically seen as mostly automatic [10], viewing seems to involve more overt choices about which webpages to visit or which products to view. Thus, we conceptualize viewing as a slower and more controlled process than gaze, which allows people to gaze—more automatically—if, when, and where they wish. This type of distinction, in and of itself, is not new: scholars since at least Yarbus [27] have noted the importance and interdependence of automatic and controlled varieties of visual attention. Yet, empirical research on the duration of people’s gaze, especially in the decision-making domain, has appeared to focus more on automatic processes. Thus, although related principles may govern viewing and gazing, a separate investigation of viewing remains theoretically important. In addition, the clickstream data that many websites actually collect would seem to correspond more closely to viewing than gazing [17], as it indicates how long consumers spend on webpages but not where they gaze. Thus, our findings on viewing could have immediate practical relevance.

## Gazing

Building on Yarbus [27], recent research has shown that people tend to spend more time gazing at an item that they will later choose than at items that they will later reject [8–10]. The source of this gaze bias, however, remains elusive, with research focusing on whether gaze reflects what people like [10] or what they plan to choose [8]—categories that can diverge when preferences and pragmatic considerations diverge, as they often do in purchasing decisions.

Early studies of gazing asked people to choose which of several items they liked most [10, 28–29], suggesting that people look at what they like (preferential looking) [30–31] and like what they look at (mere exposure) [32–33]. These simultaneous processes produce a positive feedback loop that has been shown to produce a precipitous gaze shift toward a chosen item about 1.6 seconds before the choice, i.e., a gaze cascade. This cascade ultimately means that people look longer at items that they will choose, i.e., they exhibit a gaze bias. Because both preferential looking and mere exposure involve liking, and because a gaze cascade is sometimes more pronounced for preference than non-preference decisions, Shimojo and his colleagues concluded that a person’s gaze indicates what they like [10, 28–29]. In other words, gaze reveals people’s most-preferred decision alternative.

Other research, however, suggests that gaze indicates what people will choose rather than what they like—two categories that happen to coincide when people are asked to choose what they like but may not coincide when other considerations are important [8, 24, 34–35]. Studies that support this conclusion have documented a gaze bias in non-preference decisions by asking, for instance, which of several photographs was taken more recently [24]. They have also observed a gaze bias that does not result in a gaze cascade [8, 35], suggesting that people gaze to encode decision-relevant information, possibly but not necessarily because they like the item. According to this perspective, people gaze to encode information; when asked to make a preference decision, this also signals and coincides with what they like. Thus, this perspective suggests that encoding and liking processes are not mutually exclusive.

Schotter et al. [9] attempted to integrate these perspectives by suggesting that encoding is the “master” mechanism behind the gaze bias and that liking is a separate process that also directs people’s gaze during preference decisions. According to this perspective, people primarily gaze at items to encode the information needed to make a choice [8, 24], but their gaze also automatically gravitates toward the item they like the most[30]. When people are making decisions solely on the basis of their preferences, the encoding and liking processes combine to direct attention toward a single item, and a significant gaze bias emerges [10, 29–30]. Schotter et al. [9] documented this effect and also eliminated it by including an experimental condition that asked people to select which of two items they *disliked*. As predicted, the dislike decision attenuated the gaze bias: encoding processes directed people’s gaze toward the most disliked item, while liking directed their gaze toward the most-liked item, i.e., the two processes pulled attention in different directions.

Overall, then, Schotter et al. suggest that encoding and liking are two separate mechanisms that can, but need not direct gaze to the same item. Whether they are additive, subtractive, or otherwise related depends on the nature of a person’s decision. Ironically, perhaps, this perspective is consistent with some of the earliest work on visual attention [27], which documented that slight differences in task instructions can lead to dramatic differences in people’s patterns of eye fixation. Thus, the Schotter et al. perspective returns full-circle some of the earliest thinking on visual attention.

In addition, Schotter et al.’s focus on the nature of the task raises critical new questions for purchasing decisions, which naturally involve elements that decision-makers like (e.g., features of the purchase item) and dislike (often but not always the price). In particular, purchasing decisions often ask decision-makers to act on both their preferences (what they “like”) and any pragmatic concerns like price that prevent them from indulging their preferences (and which they accordingly “dislike”). Determining whether visual attention reveals what people like or what they will purchase, then, becomes particularly interesting when prices are unsupportive of a person’s preferences, i.e., when a preferred item is costly and a non-preferred item is not.

Although Schotter et al. investigated gazing in liking as well as disliking decisions, research that considers visual attention in decisions that involve liking and disliking at the same time (like purchasing) remains thin. In particular, research on visual attention with prices [20] has not typically tried to disentangle encoding and liking processes, making it hard to determine whether viewing indicates preferences. Thus, scholars continue to call for research that puts prior findings into an economic context, e.g., “investigating the role that visual attention plays in stimulating sales would fill an important gap in current knowledge” [36].

## Viewing Time and Purchasing

A close examination of clickstream data [12, 17] reveals that people often initiate the purchasing process by exploring what is available and what they might like, with little intent to purchase. Only later, and only in some cases, do they return to seriously consider prices and make a purchasing decision. This may be especially true of “big-ticket” purchases like cars or vacations in which substantial research is needed and prices may depend on final specifications. Recognizing that this two-step process characterizes some but not all viewing behavior, our experiments modeled it by first allowing people to view items without prices, and then asking them to consider these same items with prices. In addition to simulating the above-noted viewing and purchasing process, this procedure allowed us to establish a baseline measure of viewing time and to experimentally manipulate the relationship between prices and original, subjective preferences, facilitating clearer inferences about the relationship between viewing time and preferences.

As noted, people tend to display a gaze bias for preference decisions without prices. We introduce a related concept, a “viewing bias,” in which people may choose to spend more time viewing their most-preferred than less-preferred items. In addition to providing a baseline for subsequent hypotheses, documenting this bias could indicate whether visual biases extend from relatively automatic processes like gazing to relatively controlled processes like viewing, i.e., whether relatively overt “System 2” viewing processes [37] are also open to bias. As also noted, the *a priori* focus of the present research was on the information that viewing time might provide about people’s most-preferred option—their “favorite” option—relative to other less-preferred alternatives. Thus, our model started with the admittedly basic but nevertheless foundational prediction that in the absence of any price information, viewing, like gazing, would reveal people’s most-preferred option:

*Hypothesis 1: Without prices, decision-makers will exhibit a viewing bias, i.e., they will spend more time viewing their most- rather than less-preferred items.*

The inclusion of prices prompts decision-makers to consider the economics of their possible choices, altering what might have otherwise been a purely preference-driven decision [38]. In other words, economic factors push people to determine whether their attraction to an item is sufficient to warrant the associated monetary expenditure, especially when the most-preferred item costs more than less-preferred items, i.e., when prices are unsupportive of preferences. This is the situation that several conditions in our studies simulated: after asking people about their preferences (and assessing their viewing times unobtrusively), we gave their most-preferred item a high price and their least-preferred items lower prices. Since they had already indicated that they liked what was now the most costly item, we expected that price information would alter their preferences and purchase intentions as well as the basic attentional processes supporting their decision-making.

*How* would prices impact viewing time, though? We expected the impact of prices to depend on their extremity—especially whether a preferred item was moderately or extremely costly, thereby triggering a decision that was more similar to a “like” or “dislike” decision [9], respectively. To illustrate, consider a Ferrari, which many people prefer but few can presumably afford because of its extreme price. When comparing a Ferrari with a more reasonably priced car like a Honda, people’s preferences will not always match their ultimate purchase: Even if they prefer the Ferrari and relish the opportunity to view its attractive features, they will probably not buy it. Instead, they will probably buy the Honda, meaning that the item that attracts their viewing for preferential reasons (the Ferrari) is not the item that attracts their viewing for encoding reasons (the Honda). This situation—a liked item pulling visual attention in a different direction than an ultimately chosen item—conceptually resembles Schotter et al.’s dislike decisions.

Thus, in purchasing decisions where the preferred option costs a great deal, (i.e., when prices are “extremely unsupportive” of preferences), we predict that people’s viewing time will split among multiple items. Specifically, they should view their most-preferred but most expensive item for liking reasons, but their less-preferred but more affordable items for encoding reasons—which will collectively attenuate the viewing bias. Furthermore, if prices are truly “extremely unsupportive,” their purchase intentions (the item they say they “prefer” after knowing prices) should change. Thus, in direct opposition to the viewing bias predicted in Hypothesis 1:

*Hypothesis 2: Extremely unsupportive prices will reduce the viewing bias, causing people to split their viewing time between the most-preferred/most expensive item and the less-preferred/less expensive items, leading them to intend to purchase a previously less-preferred item.*

At the other extreme, fortunate consumers might find their most-preferred item with a low price (i.e., prices are “supportive” of preferences). This might occur, for example, when a preferred item goes on sale. In this situation, prices add little new information because a decision-maker’s purchasing choice is obvious. Since they already know which item they like and intend to purchase, they have almost no need to encode additional information. This gives them the opportunity to indulge their epistemic motivations by browsing non-preferred alternatives, i.e., seeking novelty [12]. Thus, we predict that supportive prices will also dampen the viewing bias:

*Hypothesis 3: Supportive prices will reduce the viewing bias, causing people to split their viewing time between the most-preferred/most affordable and less-preferred/less affordable items, even though they will intend to purchase the most-preferred item.*

Oftentimes, however, people make purchasing decisions among a set of items that have neither supportive nor extremely unsupportive prices: they are not lucky enough to find inexpensive items that they like better than expensive items, and they never consider buying a Ferrari. Because price and quality are often closely connected, prices and preferences often increase at a similar pace. A car-buyer might prefer the slightly more-expensive Honda Accord to a Honda Civic, for example, but the price difference between them is relatively moderate (and far less extreme than the price difference between any Honda and a Ferrari). In these more common situations, when a preferred item costs only somewhat more than less-preferred items (“moderately unsupportive” prices), preferences have a better chance of predicting purchase intentions than they do when prices are extremely unsupportive: people are more likely to purchase a preferred Accord over a non-preferred Civic than a preferred Ferrari over a non-preferred Civic. In other words, under moderately unsupportive prices (e.g., a choice among the Hondas), the ultimately purchased item (e.g., the Accord) is likely to attract viewing for both preferential and purchase encoding reasons—a situation that resembles Schotter et al.’s [9] “like” decision as well as Shimojo’s original research [10, 28–29]. Thus, in contrast to the attenuated viewing biases predicted by Hypotheses 2–3, we predict that a viewing bias will still emerge under moderately unsupportive prices:

*Hypothesis 4: With moderately unsupportive prices, decision-makers will exhibit a viewing bias, spending more time viewing their most-preferred/most-expensive item than less-preferred/less-expensive items, and they will intend to purchase the most-preferred item.*

Table 1 summarizes the hypotheses. What kinds of prices are extremely versus moderately unsupportive? We acknowledge that this is a difficult question that depends on characteristics of both the decision-maker and the decision, i.e., one person’s moderate price may be another’s extreme price. Our research addresses this challenge by treating “extremely” versus “moderately” as relative rather than absolute terms, making our predictions probabilistic and our empirical tests conservative—a strategy that could readily be extended to real-world decisions.

**Table 1:** Predictions.

**Prices**	**Hypothesis**	**Is a viewing bias present?**	**Do purchase intentions match preferences?**
None	1	Yes	—
Extremely Unsupportive	2	No	No
Supportive	3	No	Yes
Moderately Unsupportive	4	Yes	Yes

## Study 1

Study 1 tested Hypotheses 1 and 4 by examining whether preferences predicted viewing time—first without prices, then with moderately unsupportive prices—as well as whether preferences predicted purchase intentions. Participants viewed and recorded their preferences for four commercially-available posters. They then viewed the posters again in a purchasing context, with moderately unsupportive prices.

### Methods

**Participants.** One-hundred forty-two undergraduates from a business school participant pool (44.23% male; from 18 to 22 years old; *M =* 19.67, *SD =* 1.16) at a major university in the Midwest U.S. participated in a 30-minute experiment on “decision-making.” They each received a participation fee of $8. Two participants were excluded for not looking at one or more of the items in the initial viewing period.

**Ethics statement.** This study was approved by the IRB at Northwestern University. Written consent was obtained before the study.

**Design.** In this study, participants viewed four images of posters (numbered 1 through 4). We counterbalanced the association of number and image using a Latin-square design [39]. Thus, participants in the study were randomly assigned to four conditions, corresponding to four different number-image combinations.

**Procedures.** Participants sat at a computer monitor in a private room or cubicle. They were told to look at each of the four poster-images for as long as they liked (see S1 Appendix). The screen that presented these instructions also displayed a diagram of a keyboard and demonstrated how participants could select and display any of the four items. The next screen reiterated these instructions and asked them to press one of the numbers to see their first item. Viewing time for each page was measured as the total time that they spent viewing it. We acknowledge and even emphasize that viewing time does not necessarily correspond exactly to the time spent gazing at the items, as this follows from our distinct focus on viewing.

Each of the four images was a poster-style depiction of a famous painting, displayed in the center of the screen, using approximately 50% of the screen’s space (see S1 Appendix). Empty, black space surrounded each image; its number (e.g., “Image 1”) appeared below it. A set of instructions appeared above it: “Press 1, 2, 3, or 4 to view the posters. When you’re finished, press the <SPACEBAR> to see a blank screen.” Because pretesting indicated that a few participants wanted to look at the posters for more than a minute, the program actually allowed them to look at the images for a total of 90 seconds. We acknowledge that participants may not have been particularly interested in purchasing these specific posters, but that makes our test for the effect of preferences on viewing conservative.

When participants pressed the spacebar, the words “BLANK SCREEN” filled the center of the screen. If participants did not return to any of the items within ten seconds, or after 90 seconds had elapsed, a new screen asked them to rank the images in terms of their preferences. To do this, they saw a screen with four, very small (~.50 cm) thumbnails of each item that were vertically stacked in random order on the left side, plus the words “Please rank the images of posters from Most-preferred to Least-preferred by dragging them to the right side of the screen.” On the right side, the words “Most-preferred” and “Least-preferred” appeared in large letters at the top and bottom, respectively. Participants dragged and stacked the images to record their preferences. The instructions then indicated that they would now see the posters again but with their actual prices, that all four posters were the same quality and size, and that they should examine the posters as if they were about to purchase one (see S1 Appendix).

**Prices.** Participants saw the same four images in the same order, with a price in large, white text below each image. Based on pretesting in this same population, the following prices were identified as moderately unsupportive: Their most-preferred poster was priced at $12; their next most-preferred poster’s price was $9; the next, $6, and the last, $3. As before, they could view each image as long as they wished within the viewing period. Participants then ranked the posters, with their prices, according to their preferences, considering both “appearance and price.” Participants concluded the experiment by answering some post experimental questions (see below).

### Analyses and Measures

**Overview.** This and all of our remaining studies tested three types of predictions. First, we sought to determine whether people’s preferences influenced the amount of time that they viewed the items before prices were introduced (“pre-price viewing times”; Hypothesis 1). Second, we examined whether their preferences influenced the amount of time that they later viewed the same items with prices attached (“post-price viewing times”; first part of Hypotheses 2–4). Third, we examined whether their preferences predicted their purchase intentions (second part of Hypotheses 2–4).

**Preferences and pre-price viewing times.** In this first set of analyses, preferences served as the independent variables, and pre-price viewing times served as the dependent variables. Preferences were operationalized as participants’ preference rankings of the thumbnails, with the item that they ranked as #1 defined as their most-preferred, #2 as their second-most-preferred, etc. Pre-price viewing times were initially measured as the total time that participants viewed each image (in milliseconds) during the initial, without-price viewing stage. If participants looked at an image several times, the times from each viewing were summed. Raw viewing times were transformed into the percentage of each participant’s total viewing time spent on each image. Because there were four images, the average (baseline) percentage is 25%. In accordance with Hypothesis 1, these initial analyses were conducted using repeated-measures Analyses of Variance (ANOVAs) with one factor (preferences), consisting of two levels (item ranked #1 versus items ranked #2–4).

Since people cannot logically express any preferences for visual images until they have seen them, preferences were necessarily measured after pre-price viewing time. Our assumption, grounded in economic theory, however, was that people have stable, *a priori* preferences, even if they cannot yet record them in a particular instance. In other words, we assumed for these basic and initial analyses (only) that the independent variable theoretically preceded the dependent variable, even if we could not methodologically record it before the dependent variable.

**Preferences and post-price viewing times.** In this next set of analyses, preferences (defined in the same way as above) served as the independent variables, and post-price viewing times served as the dependent variables. Post-price viewing times were initially measured as the total time that participants viewed each image (in milliseconds) during the second, with-price viewing stage. These times were then summed and transformed into percentages in the same way as above. Following Hypotheses 2–4, these analyses were conducted using repeated-measures ANOVAs with one factor (preferences), consisting of two levels (item ranked #1 versus items ranked #2–4).

**Preferences and purchase intentions.** In this final set of analyses, preferences (defined in the same way as above) served as the independent variables, and purchase intentions served as the dependent variables. Purchase intentions were defined as participants’ rank-ordering of the images after seeing prices (and explicit instructions to consider prices), reflecting the fact that “preferences” stated after seeing price information naturally incorporate that information can no longer be considered pure indicators of preference for the item itself. Following Hypotheses 2–4, these analyses were conducted using repeated-measures ANOVAs with one factor (preferences), consisting of two levels (item ranked #1 versus items ranked #2–4). As a check on these results, we also computed the Spearman correlation between preferences and purchase intentions.

**Post-experimental reflections.** Across studies, participants answered a similar set of post-experimental questions (see S2 Appendix), which probed for suspicion and assessed participants’ general interest and engagement, how long they thought they looked at each image, their reactions to the prices, and how much they would pay for each poster. A final question informed participants that we hoped to distribute copies of the actual posters in exchange for $3. Were they interested? If so, which poster?

### Results

On average, participants viewed each non-priced item for 12.32 seconds (*SD =* 4.87), with an average of 3.47 views per item (*SD =* 2.42). Viewing times dropped in the second, with-price round (*M =* 6.49 seconds, *SD =* 3.78), with an average of 4.00 views per item (*SD =* 2.94). Order of presentation mattered, as participants generally viewed the item associated with number 1 longer than the items associated with other numbers (all *p’s <* .05). Item order, however, did not significantly influence participants’ preferences for the items (all *p’s >* .14).

**Figure 1:**
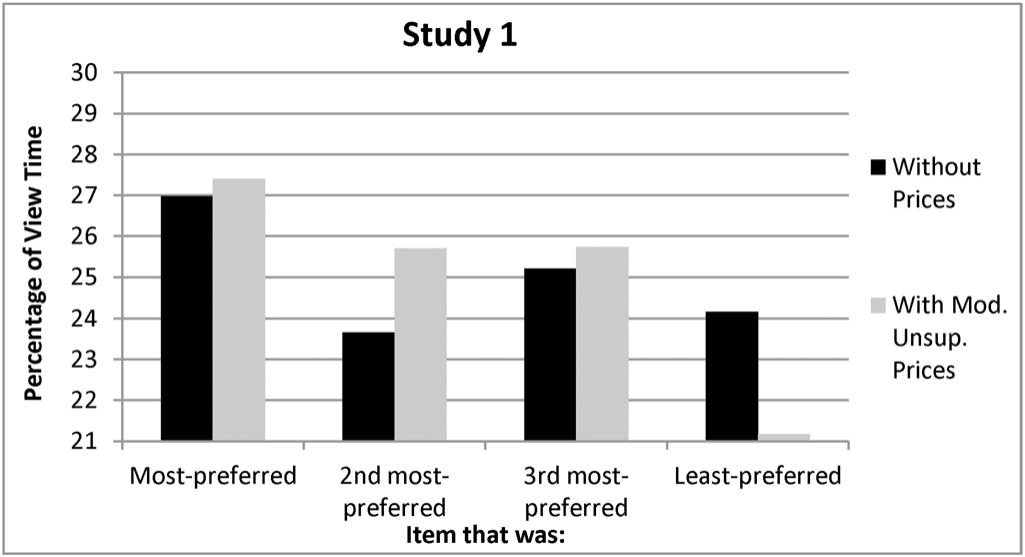
Percentage of Without-Price and With-Price Viewing Time Allocated to Each Item in Study 1.

Viewing times indicated the presence of a viewing bias, supporting Hypothesis 1: participants viewed their most-preferred item longer than they viewed the average of the other three items, *F(*1, 141) = 4.78, *p =*.03, before prices were introduced (see Fig. 1).

We tested our data for consistency with the assumptions of repeated-measures ANOVA. It was consistent with most of these assumptions. We tested the normality of residuals assumption by producing Q-Q plots and running Shapiro-Wilk tests. Although the Q-Q plots did not indicate substantial divergence from the straight line, suggesting that any deviations from normality were not severe, the Shapiro-Wilk tests did indicate that the assumption of normality could not be maintained. Given the Q-Q plots and the robustness of repeated-measure ANOVAs to the normality assumption, we do not regard these results as threatening our conclusions, but we believe that it is important to state this result explicitly.

**Figure 2:**
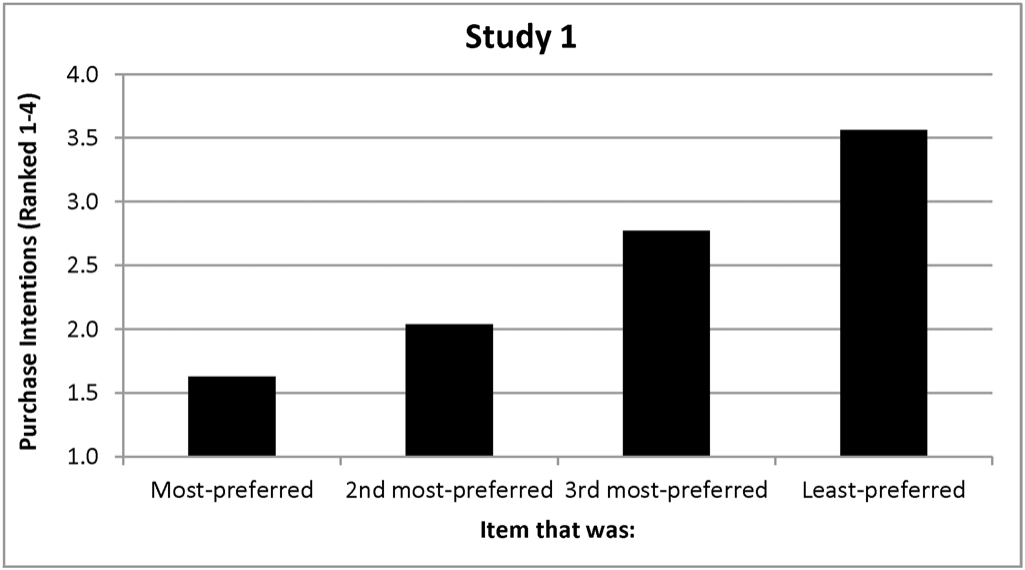
Purchase Intentions as a Function of Preferences in Study 1.

Hypothesis 4 predicted that the viewing bias would still emerge under moderately unsupportive prices. It did, as participants again viewed their most-preferred item longer than the other three items even after moderately unsupportive prices were introduced, *F(*1, 141) = 8.88, *p =* .003 (see Fig. 1). Hypothesis 4 also predicted that people would intend to purchase their most-preferred item under moderately unsupportive prices. They did, continuing to rank their originally-most-preferred item higher than the other three items, *F(*1, 141) = 163.79, *p <* .001, which caused preferences and purchase intentions to correlate (Spearman correlations for the four items = .50,.67,.66, and .73, all *p’s <* .001; see Fig. 2). This all supports Hypothesis 4 and its contention that a viewing bias would emerge under moderately unsupportive prices, which alter neither viewing time nor purchase intentions.

An alternative explanation is that people ignored or failed to notice the prices. The data argue against this interpretation, however, as the addition of prices led people to spend more time (25.7% versus 23.7%) viewing the item that they had originally ranked second, suggesting that they were now considering whether its lower price might justify changing their preferences. This was supported by a repeated-measures ANOVA with post-price viewing times for the #2 item as the dependent variable and one factor (round) consisting of two levels (pre- vs. post-price) as the independent variable, *F(*1, 141) = 4.33, *p =*.04. Participants also spent less time (21.1% versus 24.2%) viewing the item that they had originally preferred least, suggesting that this item’s low price ($3) did not increase its appeal, as indicated by a similar ANOVA on viewing times for the least-preferred item, *F(*1, 141) = 5.01, *p = *0.03. Parallel ANOVAs indicated that viewing times for the items previously ranked most- and third-most-preferred were similar with and without prices (*p’s >* .60). This all suggests that people paid attention to the prices but did not alter their choices, as Hypothesis 4 predicted.

None of the participants indicated that they suspected that the experiment was about their viewing behavior; when asked, however, they did realize that they viewed their most-preferred more than their least-preferred poster—both without (*M’s =* 38.94% vs. 21.48%; *SD’s =* 20.13, 14.38; *F(*1, 141) = 95.29, *p <*.001) and with prices (*M’s =* 33.59% vs. 18.94%; *SD’s =* 18.35, 13.35; *F(*1, 141) = 80.25, *p <*.001). They overestimated their actual viewing proportions, however (without prices: 26.98% and 24.16%; with prices: 27.75% and 21.77%), suggesting that they were aware but not perfectly aware of their viewing behavior.

Participants indicated that they would pay about $7.42 more for their most-preferred (*M =* $8.80, *SD =* 2.22) than for their least-preferred poster (*M =* $1.38, *SD =* 1.44), *F(*1, 141) = 442.54, *p <*.001)—a difference that approximates the $9 price span ($12-$3) that they had just seen. This supports our definition of these prices as moderately unsupportive. Finally, 81 participants (57.04%) indicated that they would like to purchase a poster for $3 (see Table 2). Of these, a plurality (49.38%) selected the poster that was both originally and ultimately preferred—consistent with the prediction that preferences would remain relatively stable under these price conditions. Some participants, however, selected the poster that was only their originally- (25.93%) or ultimately-most-preferred (18.52%); only four (6.17%) selected a poster that they never ranked as their most-preferred.

**Table 2:** Selecting a Poster for $3.

	***Among all participants***	***Among those who wanted a poster,*% whose selection was:**			
**Prices**	**% who wanted a poster**	**Both their original and their ultimate preference**	**Their original but not their ultimate preference**	**Their ultimate but not their original preference**	**Neither**
*Study 1:*Moderately unsupportive	57.04%	49.38%	25.93%	18.52%	6.17%
*Study 2:*Moderately unsupportive	52.33%	62.22%	22.22%	13.33%	2.23%
*Study 3:*Extremely unsupportive	50.00%	8.82%	35.29%	26.47%	29.42%
*Study 3:*Moderately supportive	48.48%	31.35%	12.50%	0.00%	56.15%
*Study 4:*Moderately unsupportive	18.18%	77.27%	0.00%	9.09%	13.64%
*Study 4:*Extremely unsupportive	27.43%	51.61%	19.35%	12.90%	16.13%
*Study 4:*Moderately supportive	27.73%	81.80%	12.10%	0.00%	6.10%
*Study 4:*Extremely supportive	31.19%	67.65%	11.76%	0.00%	20.59%
*Study 4:*Moderate-random	23.14%	67.86%	21.43%	0.00%	10.71%
*Study 4:*Extreme-random	24.00%	66.67%	20.00%	3.33%	10.00%

### Discussion

These results present basic evidence for the existence of a viewing bias: decision-makers chose to spend more time viewing more-preferred items (supporting Hypothesis 1). The results also begin to illuminate the impact of prices on viewing. When a preferred item had a high but not outrageously high price (i.e., moderately unsupportive prices), the viewing bias still emerged: people viewed their most-preferred item more than the other items, and about as much as they did when it did not have a price (in relative terms; supporting Hypothesis 4). They also continued to prefer that item despite its price. Thus, although they seemed to notice the prices, their viewing and purchasing behavior did not change, suggesting that moderately unsupportive prices do not push decision-makers’ viewing away from their preferences. Put differently, viewing time appears to provide insight into preferences under moderately unsupportive prices.

Study 1 was a first investigation of viewing time and preferences in the context of prices and potential purchases. Study 2 tested the same hypotheses with a different set of stimuli and different procedures, adding a practice round to (potentially) minimize order effects.

## Study 2

Study 2 increased the items’ variety by including posters that differed in style and type, i.e., a painting, a landscape, a cityscape, and a car (see S1 Appendix). It also added a practice round that allowed participants to become acquainted with the technology and viewing procedure.

### Methods

**Participants.** Eighty-six different undergraduate students (35.29% male, from 18 to 22 years old; *M =* 19.47, *SD =* 1.19) from the same participant pool participated for $8 each. One participant who did not view all of the images at least once in the initial viewing period was dropped.

**Ethics statement.** This study was approved by the IRB at Northwestern University. Written consent was obtained before the study.

**Design, procedure, analyses, and measures.** This study used the same design as the prior study. It also used the same basic procedures, prices, and price assignments as Study 1, with a few small changes. In a new practice round, participants viewed four colored shapes to familiarize themselves with the procedure before moving to the experimental sessions, during which they viewed four new poster-images (see S1 Appendix). All analyses and measures were the same as in Study 1.

### Results

On average, participants viewed each non-priced item for 11.11 seconds (*SD =* 4.31), 3.82 separate times (*SD =* 2.47). The practice round was effective, as order effects no longer surfaced. Viewing times fell again during the second, with-price round, to an average of 6.64 seconds (*SD =* 4.42), with each item viewed an average of 3.80 separate times (*SD =* 2.12).

**Figure 3:**
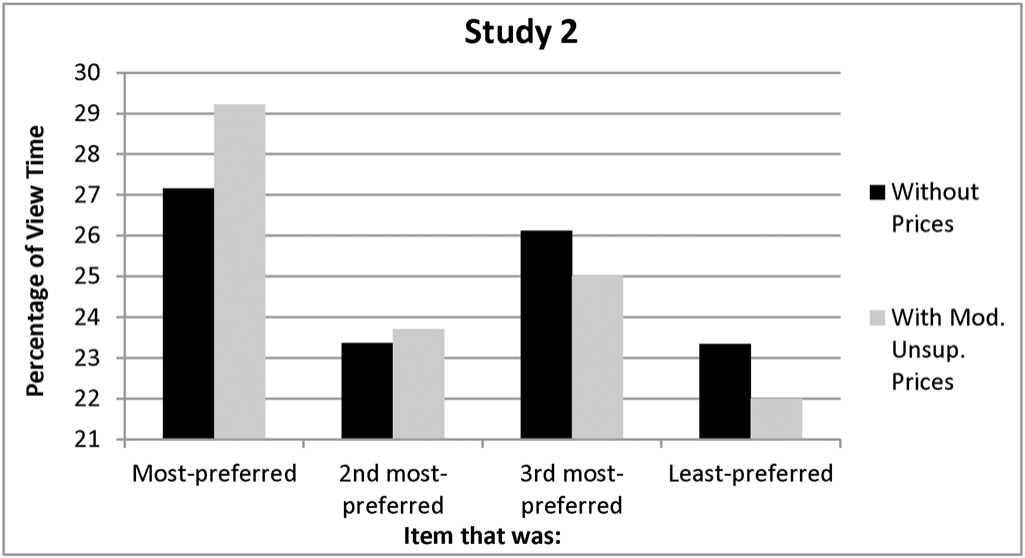
Percentage of Without-Price and With-Price Viewing Time Allocated to Each Item in Study 2.

**Figure 4:**
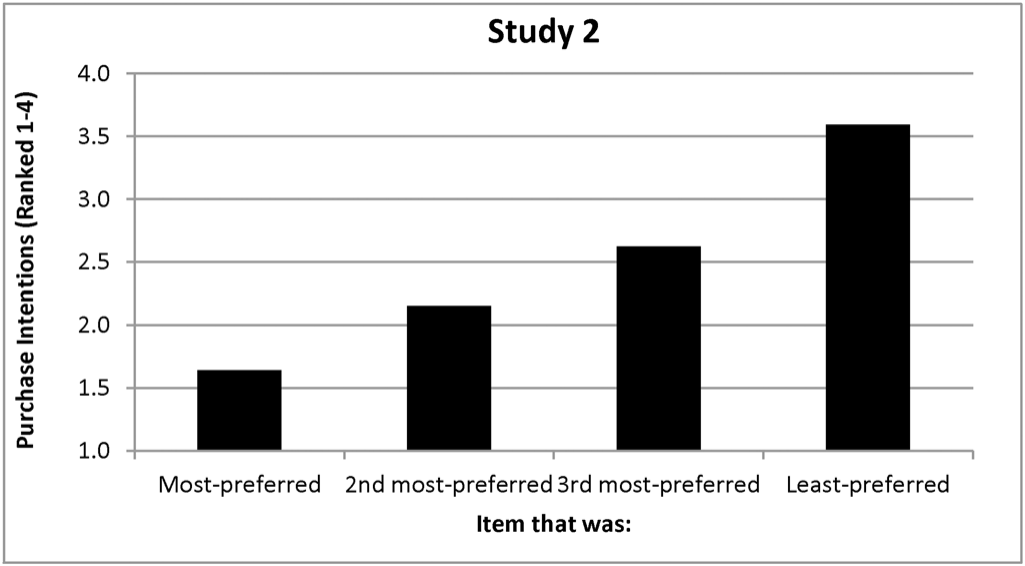
Purchase Intentions as a Function of Preferences in Study 3.

As before, participants’ pre-price behavior reflected a viewing bias: in support of Hypothesis 1, they viewed their most-preferred item longer than they viewed the other three items, *F(*1, 85) = 4.44, *p = *0.04 (see Fig. 3). Also as before, people continued to view their most-preferred item longer than the other three items after prices were introduced, *F(*1, 85) = 12.35, *p =* .001 (see Fig. 3; supporting the first part of Hypothesis 4). Additionally, participants continued to rank their most-preferred item higher than the other three items, *F(*1, 85) = 82.22, *p <*.001 ( supporting the second part of Hypothesis 4), so preferences and purchase intentions were correlated (Spearman correlations for the four items = .47,.59,.27,.62, all *p’s <* .02; see Fig. 4).

As before, participants believed that they viewed their most-preferred more than their least-preferred poster—both without (*M*’s = 42.76% vs. 19.61%; *SD*’s = 17.93, 11.48; *F(*1, 75) = 104.54, *p <* 0.001) and with prices (*M*’s = 41.05% vs. 17.24%; *SD*’s = 17.78, 11.02; *F(*1, 75) = 94.55, *p <* 0.001)—although they did not report awareness that the experiment was about viewing, and their estimates were once again relatively extreme. Participants indicated that they would pay an average of $8.98 more for their most- than for their least-preferred poster, *F(*1, 75) = 307.25, *p <* .001.

A majority of the participants (*N* = 45; 52.33%) indicated that they wanted to purchase a poster for $3 (see Table 2). A larger proportion than in Study 1 (62.22%) selected the poster that was both originally- and ultimately-most-preferred. Comparable proportions selected the poster that was only their original (22.22%) or only their ultimate (13.33%) preference; only one participant (2.23%) selected a poster that was never their most-preferred.

### Discussion

These results replicated Study 1’s with a more varied set of items and without order effects. As before, individuals had stable preferences, revealed by their viewing time, even after seeing moderately unsupportive prices. In other words, viewing time revealed preferences with or without prices (supporting Hypotheses 1 and 4). We designed Study 3 to explore the limits of these effects.

## Study 3

Study 3 investigated the effects of extremely unsupportive as well as supportive prices. In particular, it tested whether these prices would reduce the viewing bias by either pushing people’s viewing away from their most-preferred item or by pushing it toward other options that they had little intention to pursue (as predicted by Hypotheses 2 and 3, respectively). Because we did not expect differences as a function of *how* supportive a set of prices was, we used moderately supportive prices to provide comparability with the moderately unsupportive prices in Studies 1 and 2.

### Methods

**Participants.** One-hundred thirty-eight different undergraduate students (39.55% male, from 19 to 23 years old; *M =* 20.10, *SD =* 0.93) from the same participant pool participated for $8 each. Four participants were dropped for failing to view one or more pictures in the initial viewing period.

**Ethics statement.** This study was approved by the IRB at Northwestern University. Written consent was obtained before the study.

**Design.** In addition to repeating the Latin-square approach from prior studies, participants in this study were randomly assigned to one of two conditions: extremely unsupportive or moderately supportive prices. Based on pretesting, in the extremely unsupportive price condition, participants’ most-preferred poster was priced at $50; their next-most-preferred at $35; the next, $20; and the last, $5. For the moderately supportive prices, participants’ most-preferred poster was priced at $3; their next at $6; the next, $9, and the last, $12.

**Procedure.** The procedures duplicated Study 2’s except for the different prices. Since participants in both conditions (extremely unsupportive and moderately supportive prices) experienced the same procedure before seeing prices, we analyzed their pre-price data together. Separate analyses of the post-price data tested Hypotheses 2 and 3.

### Analyses and Measures

All analyses and measures were the same as in the prior studies.

### Results

**Figure 5:**
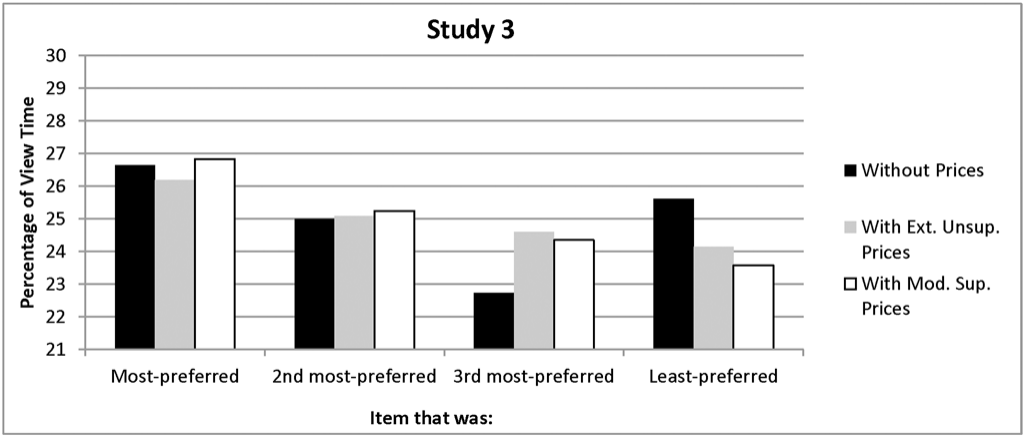
Percentage of Without-Price and With-Price Viewing Time Allocated to Each Item in Study 3.

On average, participants viewed each non-priced item for 9.96 seconds (*SD =* 4.73), 3.56 separate times (*SD =* 2.39). As in Study 2, there were no order effects. For the extremely unsupportive prices, with-price viewing averaged 6.74 seconds (*SD =* 3.98), with each item viewed an average of 3.87 separate times (*SD =* 2.45). For the moderately supportive prices, with-price viewing averaged 5.20 seconds (*SD =* 4.44), with each item viewed 3.13 times (*SD =* 2.51). As before, and consistent with Hypothesis 1, preferences predicted people’s pre-price viewing times: they viewed their most-preferred item for longer than they viewed the other three items, *F(*1, 133) = 4.94, *p = *0.03 (see Fig. 5).

**Figure 6:**
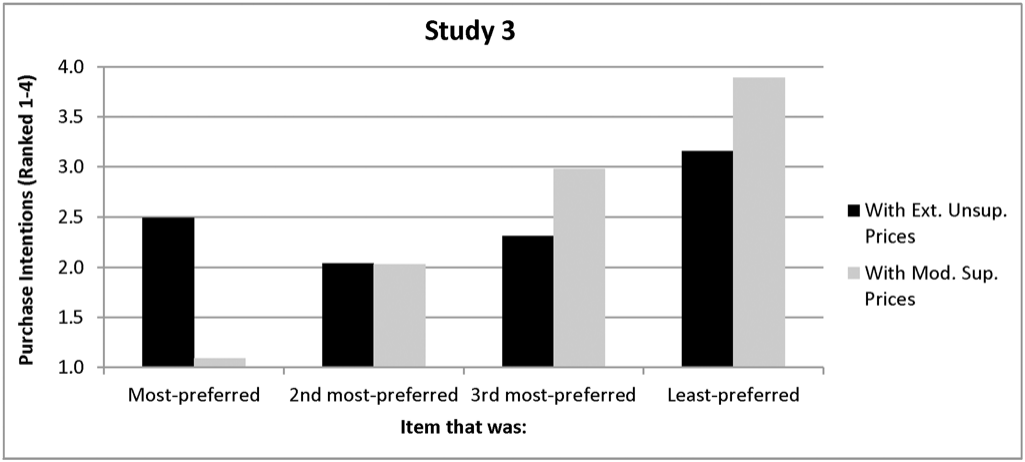
Purchase Intentions as a Function of Preferences in Study 3.

**Extremely unsupportive prices.** Extremely unsupportive prices shifted decision-makers’ viewing and changed their preferences, as Hypothesis 2 predicted. After the introduction of prices, people did not view their most-preferred item, *F(*1, 67) = 1.51, *p =*.22 any longer than the other items (see Fig. 5). Overall, a repeated-measures ANOVA with one factor (preferences), consisting of four levels (item ranked #1, 2, 3, and 4) yielded no differences in viewing times for any of the four priced items, *F(*3, 201) = .35, *p =* .79. Also as predicted by Hypothesis 2, participants facing extremely unsupportive prices did not intend to purchase their most-preferred item, *F(*1, 67) = .01, *p* = .91. Instead, 90% of the participants intended to purchase an item that was not their most-preferred (see Fig. 6). On average, people now indicated that they intended to purchase the poster that they had originally ranked second, and this preference was significantly stronger than their preference for their most-preferred item, *F(*1, 67) = 5.88, *p =* .02, and the other three items, *F(*1, 67) = 15.56, *p =* .001, as indicated by repeated-measures ANOVAs with one factor (preferences), consisting of two levels (#2 vs. #1 and #2 vs. #1, 3, and 4, respectively). Also, unlike Studies 1 and 2, preferences and purchase intentions were correlated for only two of the four items (Spearman correlations *=* .35 and.32, *p’s*< .008). The correlation was negative and significant for another of the items (Spearman correlation * = -*.24, *p =* .05) and negative but not significant for the other (Spearman correlation* = -*.22, *p* = .07). Taken together, these data provide consistent support for Hypothesis 2.

**Moderately supportive prices.** Moderately supportive prices also eliminated the viewing bias, but they maintained people’s preferences, as Hypothesis 3 predicted. Post-price, people did not view their most-preferred item, *F(*1, 65) = 1.87, *p =* .18, any more than the other items. Overall, a repeated-measures ANOVA with one factor (preferences), consisting of four levels (item ranked #1, 2, 3, and 4) yielded no differences in viewing times for any of the four priced items, *F(*3, 195) = 1.23, *p =* .30 (see Fig. 5).

As also predicted by Hypothesis 3, a large majority of the participants (83%) intended to purchase their most-preferred item instead of the other three items, *F(*1, 65) = 1142.65, *p <* .001, even though they no longer spent more time viewing it (see Fig. 6). Their preferences and purchase intentions were also highly correlated (*r’s =*.88,.94,.87,.9; all *p’s <* .001), as Hypothesis 3 suggested.

To ensure that these with-price effects were not the result of insufficient statistical power, we conducted an additional analysis of the relationship between viewing time and preferences using the full sample of 134 participants (about the same size as the Study 1 sample and more than 1.5 times the size of Study 2’s sample). The results indicated that, after seeing prices, people viewed their most-preferred item only marginally more than the other three items, *F(*1, 133) = 3.37, *p =* .07, η = .03. This effect differs from the strong effects in Studies 1 and 2 (*p*-values < .001 and η’s of.06 and.13). Because Studies 1–3 drew (different participants) from the same population and also used similar procedures, this suggests that extremely unsupportive prices and moderately supportive prices did lead people to spend less time viewing their most-preferred items.

**Post-experiment reflections.** As before, across both price conditions, participants believed that they viewed their most- more than their least-preferred poster—both without (*M*’s = 40.70% vs. 19.61%; *SD*’s = 19.21, 13.19; *F(*1, 128) = 104.50, *p <* .001) and with prices (*M*’s = 36.67% vs. 18.61%; *SD*’s = 21.63, 13.15; *F(*1, 128) = 67.15, *p <* .001)—although they did not indicate awareness that the experiment was about viewing time. Their estimates were (again) more extreme than their actual viewing patterns, especially since their actual patterns with prices revealed no significant differences.

After seeing extremely unsupportive prices, participants indicated that they would pay about $11 more for their most-preferred ($11.32) than their least-preferred ($1.28) poster, *F(*1, 66) = 290.38, *p <* .001, far less than the $45 range and $50 maximum that they had seen. This suggests that they perceived these prices as extremely unsupportive. Thirty-four participants (exactly 50%) indicated that they wanted to purchase a poster for $3 (see Table 2). Compared to Studies 1 and 2, a much smaller proportion of people (8.82%) selected the poster that they had both originally and ultimately preferred. Instead, more people selected the poster that was only their original (35.29%) or ultimate (26.47%) preference, or a poster that was never their most-preferred (29.42%).

After seeing moderately supportive prices, participants indicated that they would pay $6.40 more for their most- ($7.62) than for their least-preferred ($1.22) poster, *F(*1, 61) = 182.22, *p <* .001, suggesting that they saw these prices as supportive. Thirty-two participants (48.48%) indicated that they wanted to purchase a poster for $3 (see Table 2). As in the extremely unsupportive condition, however, relatively few participants facing supportive prices (31.35%) selected the poster that was both their originally- and their ultimately-most- preferred. Most often (56.15%), they selected a poster that was never their most-preferred; occasionally (12.50%), they selected a poster that was only their original preference. No participant selected the poster that was only their ultimate preference. It is also noteworthy that the proportion of people who selected their originally-most-preferred poster (regardless of whether it was ultimately preferred; 43.75%) is nearly identical to the proportion observed in the extremely unsupportive price condition. This suggests that, like the extremely unsupportive prices, moderately supportive prices may have reduced attraction toward the originally-most-preferred item, perhaps as a result of novelty-seeking.

### Discussion

Study 3 identified some boundary conditions for the viewing bias. Although participants viewed their preferred item longer than they viewed non-preferred items before prices were presented, their viewing departed from their preferences under either of these two pricing schemes (supporting Hypotheses 2–3). Extremely unsupportive prices seemed to push people to consider more affordable options; moderately supportive prices seemed to lead them to search for novelty and may have even led them to reconsider their preferences. In both cases, viewing wandered from their most-preferred item.

This suggests that participants in the extremely unsupportive price condition, like those in Schotter et al.’s [9] dislike condition, split their visual attention between what they liked and what they thought they should (and often did) choose. In both cases, what participants liked was not what they chose: Schotter’s participants were instructed to choose a disliked item; our Study 3 participants seemed to tell themselves to choose a less-liked but more affordable option. This systematic splitting of their viewing behavior, especially in comparison to the results of Studies 1 and 2, is consistent with a predicted mixture of liking and encoding processes. To provide a final, large-scale test of our model, we designed Study 4 to comprehensively contrast the effects under the various pricing conditions.

## Study 4

Study 4 investigated the impact of all of the supportive and unsupportive price conditions, testing all of our hypotheses in one study. It also drew from a non-student population and used a different procedure, i.e., the validated and somewhat more naturalistic Mouselab program, which made it easier for participants to view the items. The procedures also included more stimuli, randomly assigned them, and compared the focal pricing conditions against a control condition (random prices). We regarded this as a control condition because, on average, random prices bore no relationship to participants’ preferences. Our overall goal was to provide a comprehensive test of all four hypotheses in a single study, enabling direct, between-condition comparisons.

### Methods

**Participants.** Participants were 354 adults from across the U.S. (59.32% male, from 18 to 75 years old; *M =* 31.89, *SD =* 10.84) who had agreed to participate in short, online studies administered by Amazon MTurk for a small amount of compensation (in this case, $1). Compared to an undergraduate sample, the MTurk sample is more diverse and representative of society, and, at least as reliable [40].

**Design.** The design was a 2(Item Set: A or B) x 2(Price Extremity: moderate or extreme) x 3(Price Direction: supportive, unsupportive, or random) x 2(Order) mixed factorial—the last three factors within-subjects. Put simply, participants saw one set of four poster-images or the other (Set A or Set B), and they experienced two of six possible pricing schemes (e.g., moderately supportive, then extremely unsupportive) in a random order. The extremity and direction of the items’ prices were both manipulated as in Study 3, but this study included all six price combinations, including random prices. In the random condition, the prices took the same values as in the other conditions, but they were assigned to individuals’ original preferences in a single, discontinuous pattern that we randomly determined in advance. Thus, in the moderate and extreme conditions (respectively), the prices of the most-preferred items were $6 and $20, the second-most-preferred prices $12 and $50, the third-most-preferred prices $3 and $5, and the least-preferred prices $9 and $35.

**Ethics statement.** This study was approved by the IRB at Johns Hopkins University. Consent was obtained through an online form.

**Procedure.** The procedure was conceptually similar to Studies 2–3, but it used the Mouselab program [41], which presents webpages containing images covered by boxes. To view an image, people navigated their mouse over one of the boxes, which then revealed the underlying image. Concurrently, the program recorded how long the image was visible, i.e., the duration of the “mouseover.”

Participants first completed a practice round by viewing a single webpage with the same four shapes as in Studies 2–3, covered by boxes and arranged in a 2x2 layout that occupied about 2/3 of the screen. On the next page, they ranked thumbnails of the four shapes. Then, participants were asked to examine four poster-images as if they might purchase them (see S1 Appendix). The following page contained one of the two sets of poster-images, which differed from the items in prior studies and came from four categories: art, landscape, animals, and vehicles (see S1 Appendix). The items were randomly assigned to one of four locations in the 2x2 layout (upper left, upper right, etc.). Participants could view the items for as long as they wished, as many times as they wished, before clicking Continue. They then ranked thumbnails of the items and were randomly assigned to one of the six pricing conditions, reading that they should examine the posters as if they were about to purchase one (see S1 Appendix).

Participants then viewed the same items with the relevant prices appearing in black font immediately beneath each item. They re-ranked the items with prices, and then repeated the process with one of the five remaining pricing schemes. They then answered the same set of post-experimental questions as in prior studies, plus two asking how much time they spend on the internet and what percent of that time they spend on commercial websites (see S2 Appendix).

### Analyses and Measures

The without-price analyses were the same as in prior studies. The with-price analyses were conceptually similar to prior studies (e.g., same independent variable for preferences and same dependent variables), but they included additional independent variables to reflect the more complex design of this study. As noted, each participant saw two of the six possible pricing schemes. Analyses indicated that viewing times followed the same patterns for their first and second sets of prices (all *p*’s >.49). Thus, order was not included as a factor in the analyses, and the with-price viewing data were transformed into a between-subjects format, entering participant number as a covariate to control for potential individual differences in viewing. Thus, these analyses used mixed ANOVAs with item set as a between-subjects factor with two levels (A or B), price extremity a within-subjects factor with two levels (moderate or extreme), price direction a within-subjects factor with three levels (supportive, unsupportive, or random), preferences a within-subjects factor with two levels (ranked #1 versus ranked #2–4), and participant number a covariate. Additionally, several planned comparisons were conducted on post-price viewing time to examine the most important simple effects.

### Results

On average, participants viewed each non-priced item for 3.41 seconds (*SD =* 4.97), substantially less than in Studies 1–3, possibly because Mouselab made it easier. Order effects were mitigated by randomly assigning items to boxes. Across the price conditions, participants viewed each with-price item for 1.67 seconds (*SD* = 2.10).

**Figure 7:**
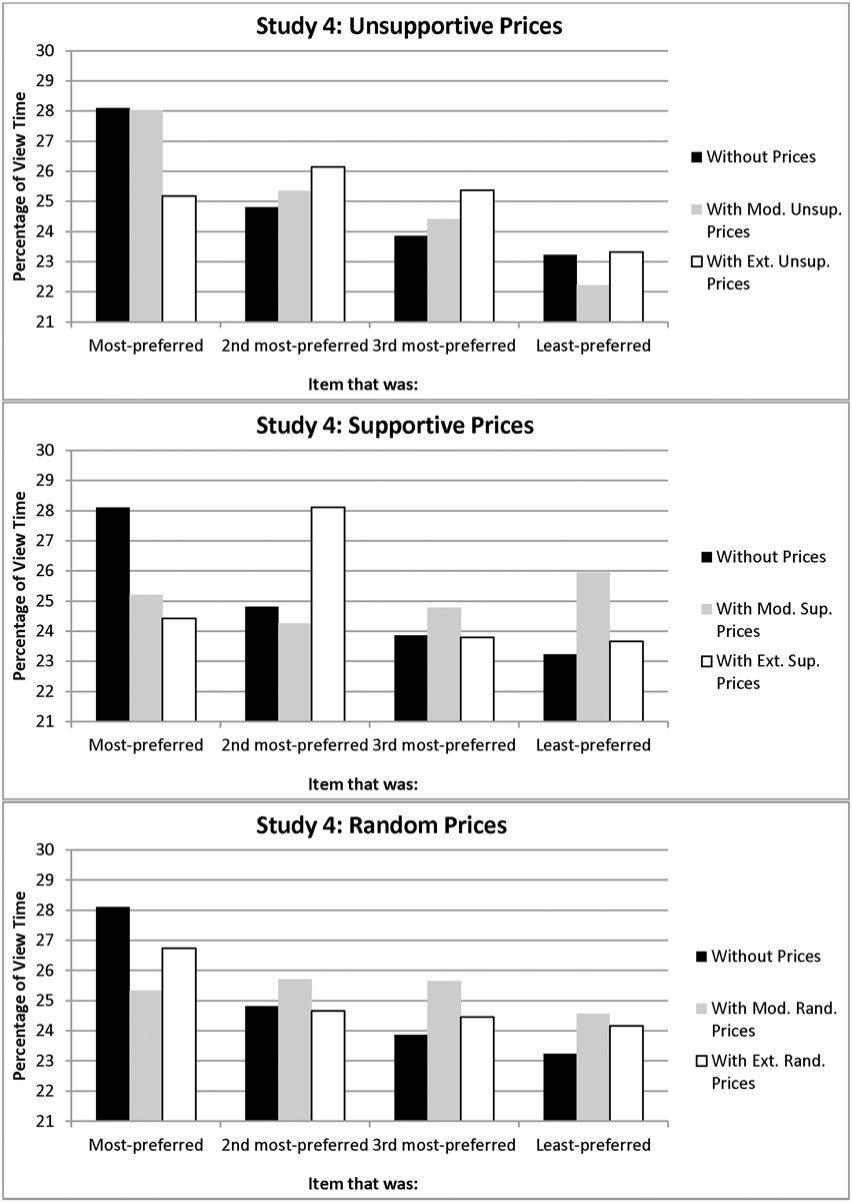
Percentage of Without-Price and With-Price Viewing Time Allocated to Each Item in Study 4 (a: unsupportive prices; b: supportive prices; c: random prices).

Unexpectedly, participants viewed the items in Set A longer than the items in Set B, and an interaction indicated that the difference in viewing time between the most-preferred and the other items was more pronounced for Set A than Set B, *F*(1, 352) = 5.91, *p* = .02. This suggests that the items in Set A were more appealing and differentiated. Nevertheless, across item sets, pre-price viewing predicted people’s preferences: they viewed their most-preferred item longer than they viewed the other three items, *F*(1, 352) = 39.29, *p*< .001 (see Fig. 7); this difference was more pronounced than in the prior studies (28.10% vs. 23.97%). This supports Hypothesis 1.

**Unsupportive prices.** Fig. 7a-7c depict viewing times across the price conditions. The viewing bias emerged for moderately unsupportive prices, but not for extremely unsupportive prices, and only the latter tended to alter people’s rankings (supporting Hypotheses 2 and 4). A mixed ANOVA on with-price viewing time for the most-preferred item versus the other three, for unsupportive prices, yielded an interaction between extremity and preferences (most- vs. less-preferred), *F*(1, 229) = 6.49, *p* = .01, indicating that the difference in viewing time between participants’ most- and less-preferred items was significantly larger for moderately than for extremely unsupportive prices (see Fig. 7a). (The analyses also yielded a main effect of extremity. We focus on the highest-order effects, both for statistical reasons and because they provide tests of our hypotheses.)

Planned comparisons of the simple effects revealed that this viewing time difference was significant for moderately unsupportive prices, *t*(120) = 3.67, *p* < .001, but not for extremely unsupportive prices, *t*(112) = .26, *p* = .80. Additional mixed ANOVAs comparing unsupportive prices to the random price control condition produced a three-way-interaction indicating that moderately unsupportive prices produced a stronger viewing bias than random prices did, but extremely unsupportive prices did not, *F*(1, 469) = 7.91, *p* = .005 (see Fig. 7c). This all supports the viewing components of Hypotheses 2 and 4.

**Figure 8:**
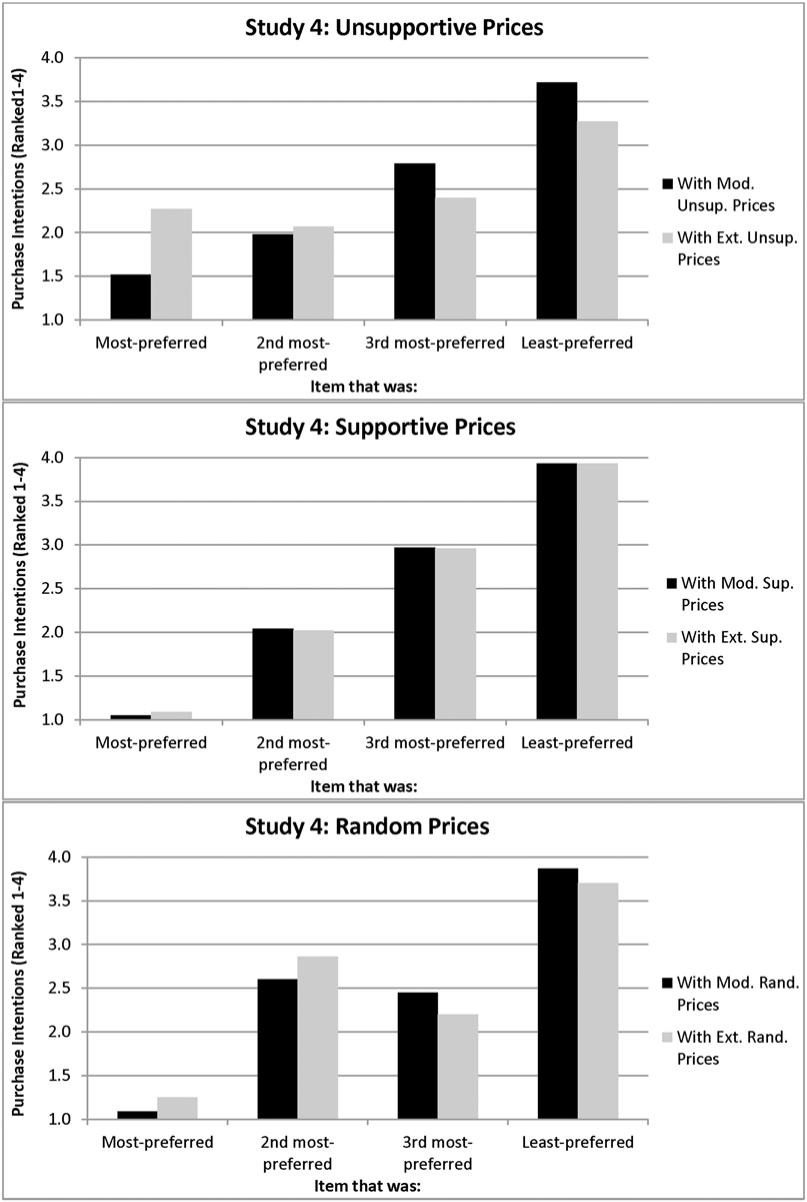
Purchase Intentions as a Function of Preferences in Study 4 (a: unsupportive prices; b: supportive prices; c: random prices).

With respect to the purchase intentions component of these hypotheses, most of the participants in the moderately unsupportive condition (67.77%) intended to purchase their most-preferred item; thus, their preferences and purchase intentions were strongly correlated (Spearman correlations for the four items = .71,.46,.63,.71, all *p’s <* .01; see Fig. 8a and 8c). This supports the purchase intentions element of Hypothesis 4.

In contrast, only 37.17% of participants in the extremely unsupportive condition intended to purchase their most-preferred item; those who switched most often elevated the item that they had originally ranked #2 to #1 (40.85%; see Fig. 8a). Thus, their preferences and purchase intentions were only correlated for two of the four items (Spearman correlations = .46,.37, *p’s <* .01); the other two correlations (.02 and.08) were not significant. These findings support Hypothesis 2.

**Supportive prices.** The viewing bias did not emerge for supportive prices, whether moderate or extreme, and people’s original preferences tended to stay the same, all of which supports Hypothesis 3. A mixed ANOVA on post-price viewing time for people facing supportive prices yielded no significant effects (all *p*’s > .18; see Fig. 7b). Also, planned comparisons of the effect of moderately and extremely supportive prices (*p*’s > .47) and comparisons of the supportive prices with the random prices (p’s > .11; see Fig. 7c) were not significant. This supports the viewing component of Hypothesis 3.

With respect to purchase intentions, almost everyone in both the moderately supportive (97.48%) and extremely supportive (94.50%) conditions intended to purchase their most-preferred item rather than the other three items. Thus, their preferences and purchase intentions were highly correlated (Spearman correlations for the four items = .91,.94,.91,.96, all *p’s <* .001; see Fig. 8b and 8c). This supports the purchase intentions component of Hypothesis 3.

**Post-experiment reflections.** Participants reported spending an average of 6.61 hours per day (*SD* = 3.36) on the internet, including 1.17 hours on commercial websites, suggesting that they were quite familiar with online purchasing. As in prior studies, and across both item sets, participants believed that they viewed their most- more than their least-preferred poster before prices were displayed (*M*’s = 49.85% vs. 20.66%), *F*(1, 332) = 481.89, *p* < .001; they also overestimated the difference and did not indicate awareness that the experiment was about viewing time. After prices were displayed, and across all pricing conditions, this same effect emerged (46.54% vs. 22.13%), *F*(1, 594) = 11.58, *p* < .001, suggesting that people have a lay intuition indicating that they allocate more of their viewing time to their more-preferred items. Thus, their intuition is only accurate when prices are either absent or are moderately unsupportive.

Participants said that they would pay $11.46 more for their most- ($14.97) than for their least-preferred poster ($3.51), *F*(1, 499) = 183.19, *p* < .001, although those exposed to extreme prices also expressed a willingness to pay more (M = $10.01) than those exposed to moderate prices (M = $8.53), *F*(1, 499) = 8.18, *p* = .004. Table 2 lists the percentage of participants in each condition who wanted to purchase a poster for $3 and, if so, which poster. Although fewer participants in this study wanted to buy a poster, those who did made choices that tended to support the hypotheses and that resembled the results of Studies 1–3. In particular, people who saw extremely unsupportive prices tended to select a poster that was their ultimate but not their original favorite, whereas participants in the other conditions tended to select their original preference.

### Discussion

Study 4 provided a comprehensive test of all four hypotheses, revealing the presence of a viewing bias without prices (Hypothesis 1) and with moderately unsupportive prices (Hypothesis 4), which did not alter people’s preferences. Thus, viewing time served as a reliable predictor of preferences under either of these conditions. For extremely unsupportive (Hypothesis 2) or supportive (moderate or extreme; Hypothesis 3) prices, however, the viewing bias disappeared, and viewing time no longer predicted people’s preferences, which changed in the former condition but not the latter conditions. Thus, these results continue to consistently highlight the same two conditions—no prices and moderately unsupportive prices—in which viewing time can provide reliable information on preferences.

By documenting all of these effects in one experiment, Study 4 provides the basis for stronger inferences than those possible from the combined results of our first three studies. Also, by documenting these same effects in a non-student sample, with a validated and more naturalistic paradigm, we have more confidence that the prior effects did not depend on their samples or methods. By including additional, randomly-assigned items as well as randomly-ordered prices, the current study also documented the robustness of these effects. In short, Study 4 provides additional evidence in support of all four hypotheses, consistently delineating the conditions that allow for the emergence of a viewing bias.

## General Discussion

When making important choices, people often balance their preferences against other, more pragmatic considerations like price. Although someone might prefer a Ferrari to a Honda, for example, their actual decision about which car to purchase is likely to incorporate information related to the two cars’ prices. This suggests that individuals’ ultimate decisions, in this example and in general, do not always reveal their preferences. Thus, identifying a person’s preferences on the basis of their decisions can be particularly challenging.

Our attempt to identify preferences took inspiration from recent research on the decision-making process [8–10]—in particular, on the information implicit in decision-makers’ patterns of visual attention. Thus, we attempted to extend the theory and data on people’s relatively automatic gaze patterns by focusing on a more deliberate process: their viewing time. Our theory suggested and results revealed that viewing time can provide important insights into people’s preferences in two, common conditions: when prices are either absent or are moderately at-odds with preferences; under these conditions, we repeatedly observed the emergence of a viewing bias. We also predicted and observed that viewing time was much less diagnostic when prices either strongly conflicted with or supported preferences; under these conditions, we did not observe a viewing bias.

In general, these results suggest that viewing can provide theoretical insights into decision-makers’ combinatorial process, shedding light on how they balance preferences and pragmatics. Gazing research has shown that people tend to fixate on what they like and/or what they plan to choose [8–10]. The current findings present critical, theoretically-relevant evidence identifying the specific conditions that may lead these processes to either converge or diverge. In purchasing situations, the results suggest that liking and encoding converge when economic considerations are manageable, e.g., when prices moderately conflict with a person’s preferences. In contrast, they diverge when economic considerations are either extreme or trivial. Thus, when more-preferred alternatives cost more but not wildly more, viewing seems to reflect preference and encoding processes that direct attention toward the same, preferred alternative. When more-preferred alternatives cost less or far more, however, viewing seems to reflect preference and encoding processes that pull attention in multiple directions, reducing attention to the preferred alternative. This suggests that the relative importance of a decision’s attractive and unattractive features may determine the relative influence of these dual processes. Future research might investigate this general implication, along with its potential boundary conditions. As one example of the latter, people may not need to spend much time viewing an item that they already know well. Documenting the effects of familiarity and other possible limits on the diagnostic value of viewing time represent important research avenues.

At a practical level, these results suggest that viewing behavior provides insight into the dynamics of individuals’ likes and dislikes. In particular, they suggest that understanding viewing time may require an understanding of prices, i.e., whether prices support or oppose preferences, and if the latter, how strongly. This suggests, for example, that Honda dealers might extract more information from the viewing behavior of the average consumer than Ferrari dealers could. In addition, car dealers might be able to extract more information during sales that make their prices moderately instead of extremely unsupportive; they might expect less, and less reliable information when their prices are extremely high or extremely discounted. These consumer-specific examples suggest a more general, practical point: the diagnostic value of viewing time may depend on the tightness of the connection between a person’s preferences and their ultimate decision. While recognizing the inherent limitations of our research, we regard these as important insights that could benefit people interested in detecting preferences—especially those with clickstream data, who could conceivably turn a 1% difference in viewing time into many millions of dollars.

Overall, this research suggests that a person’s viewing behavior can provide clues about their preferences and behavioral intentions. If moderately unsupportive prices dominate many everyday purchasing decisions, as we suspect they do, then viewing offers a tell-tale glimpse into many consumers’ inclinations. The possibility that observers might detect otherwise obscured information from an eminently observable signal offers an exciting prospect for future research.

## Supporting Information

S1 Data Study 1 Data. (SAV)

S2 Data Study 2 Data. (SAV)

S3 Data Study 3 Data. (SAV)

S4 Data Study 4 Data (between-subjects). (SAV)

S5 Data Study 5 Data (within-subjects). (SAV)

S1 Appendix Items and Instructions. (DOCX)

S2 Appendix Post-experiment Questions. (DOCX)
