# Supplementary material for: The Tell-Tale Look: Viewing Time, Preferences, and Prices
Source: PLoS One. 2015 Jan 12;10(1):e0117137. doi: 10.1371/journal.pone.0117137 (PMC4291227; doi:10.1371/journal.pone.0117137)
Supplement: S1 Appendix — (DOCX) [file pone.0117137.s006.docx]

Appendix S1. Items and Instructions

*Study 1 Instructions and Items*

You will have one minute to look at a total of four posters. You can only look at one poster at a time. However, you can look at each one for as long as you’d like (within the one minute limit), and you can return to the posters you’ve seen before. To view a poster, press the numbers 1, 2, 3, or 4 (in any order) on your keyboard. Please be sure to examine each of the posters closely. When you’re finished, press the <SPACEBAR> to see a blank screen. Otherwise, please continue to look at the set of four posters for a total of one minute by clicking the numbers.


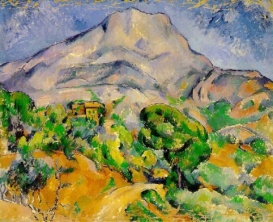

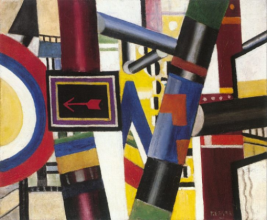

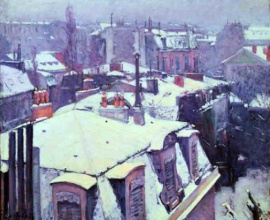

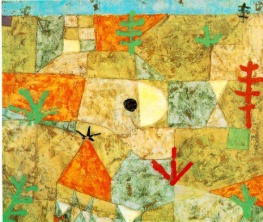


At the end of the experiment, you will be asked to make a purchasing decision about these posters. Thus, as you examine the posters and their prices, try to think about what it would be like to purchase the real posters for the prices listed.

*Study 2 and 3 Practice Round Shapes*


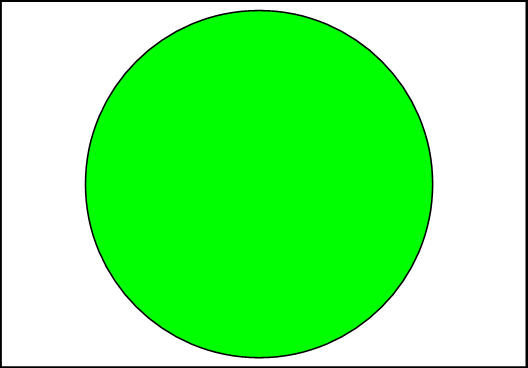

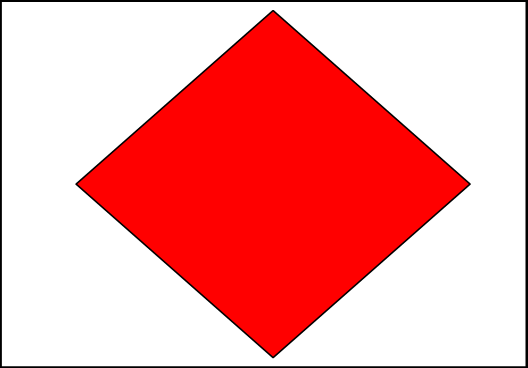

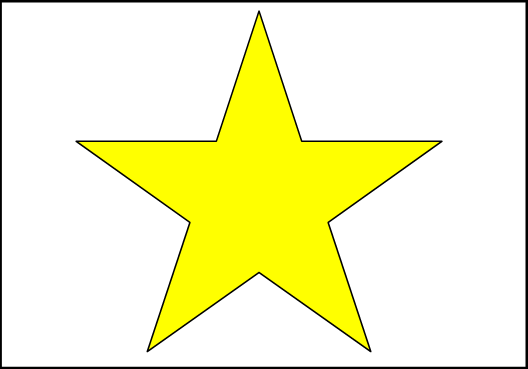

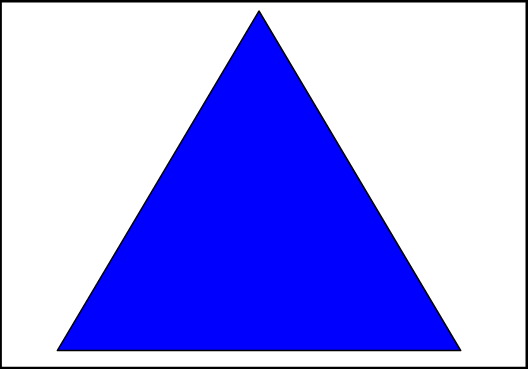


*Study 2 and 3 Items*


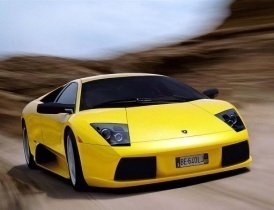

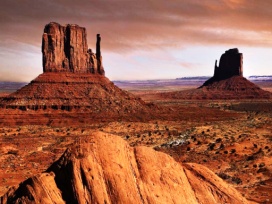

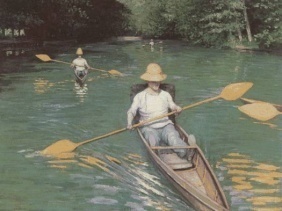

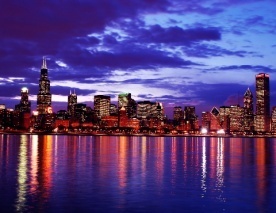


*Study 4 Instructions and Items*

On the next page, you will look at four images of actual posters that you might purchase. Please look at the images as if you were deciding which one to purchase. You may look at each poster as long and as many times as you like, but please make sure to look at each poster at least once. Whatever you do, please make sure to review the posters carefully.

Set A


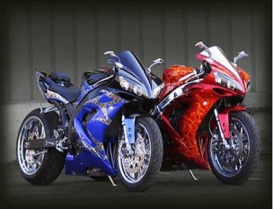

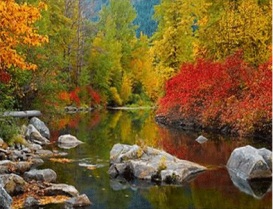

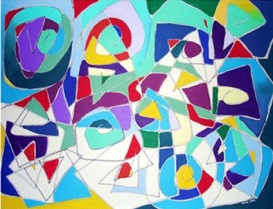

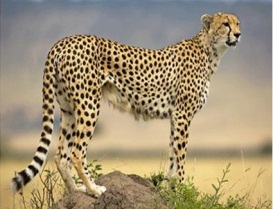


Set B


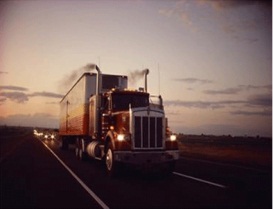

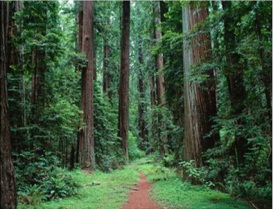

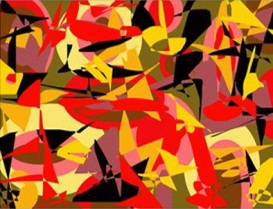

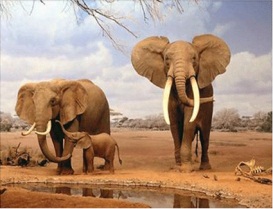


On the next page, you will look at the same four posters with prices assigned. Now that you are familiar with the four posters, please consider them in light of the following prices. Whatever you do, please make sure to review the posters and their prices carefully.
