# Supplementary material for: The Tell-Tale Look: Viewing Time, Preferences, and Prices
Source: PLoS One. 2015 Jan 12;10(1):e0117137. doi: 10.1371/journal.pone.0117137 (PMC4291227; doi:10.1371/journal.pone.0117137)
Supplement: S2 Appendix — (DOCX) [file pone.0117137.s007.docx]

Appendix S2. Post-experiment Questions

- Please tell us briefly what you think the focus of this experiment was (open-ended)
- When prices were NOT DISPLAYED, how much time did you spend examining your Most-preferred poster? (1=10%, 2=20%...10=100%)
- When prices were NOT DISPLAYED, how much time did you spend examining your Least-preferred poster? (1=10%, 2=20%...10=100%)
- When prices WERE DISPLAYED, how much time did you spend examining your Most-preferred poster? (1=10%, 2=20%...10=100%)
- When prices WERE DISPLAYED, how much time did you spend examining your Least-preferred poster? (1=10%, 2=20%...10=100%)
- About how much would you actually pay for an approximately 16" x 20" print of your MOST-PREFERRED poster? ($0, $2…$16, More than $16)
- About how much would you actually pay for an approximately 16" x 20" print of your LEAST-PREFERRED poster? ($0, $2…$16, More than $16)
- You have finished the experiment. Thank you for your participation. We are currently checking the availability of the real posters you have seen today. If we can get enough 16" X 20" prints of these posters, we are planning to offer them to participants at a discounted price of $3 (to help defray the purchase cost). If you are interested in this option and we can obtain enough prints, we will contact you by email in about two weeks. Are you interested in receiving a 16" X 20" print of one of the posters you have seen today for $3? (yes, no)
- Which poster are you interested in receiving for $3? (select one of the posters)
- Study 4 only: About how many hours per day do you spend on the internet? (open-ended)
- Study 4 only: What percentage of the time that you spend on the internet do you spend on commercial websites (i.e., websites where you could buy something)? (0%, 10%, 20%...100%)
